# Serologic Evaluation of Healthcare Workers Caring for COVID-19 Patients in the Republic of Korea

**DOI:** 10.3389/fmicb.2020.587613

**Published:** 2020-11-20

**Authors:** Jae-Hoon Ko, Ji Yeon Lee, Hyun Ah Kim, Seung-Ji Kang, Jin Yang Baek, Su-Jin Park, Miri Hyun, Ik Joon Jo, Chi Ryang Chung, Yae-Jean Kim, Eun-Suk Kang, Young Ki Choi, Hyun-Ha Chang, Sook In Jung, Kyong Ran Peck

**Affiliations:** ^1^Division of Infectious Diseases, Department of Medicine, Samsung Medical Center, Sungkyunkwan University School of Medicine, Seoul, South Korea; ^2^Division of Infectious Diseases, Department of Medicine, Keimyung University Dongsan Hospital, Keimyung University School of Medicine, Daegu, South Korea; ^3^Division of Infectious Diseases, Department of Internal Medicine, Chonnam National University Medical School, Gwangju, South Korea; ^4^Asia Pacific Foundation for Infectious Diseases, Seoul, South Korea; ^5^College of Medicine and Medical Research Institute, Chungbuk National University, Cheongju, South Korea; ^6^Department of Emergency Medicine, Samsung Medical Center, Sungkyunkwan University School of Medicine, Seoul, South Korea; ^7^Department of Critical Care Medicine, Samsung Medical Center, Sungkyunkwan University School of Medicine, Seoul, South Korea; ^8^Division of Infectious Diseases and Immunodeficiency, Department of Pediatrics, Samsung Medical Center, Sungkyunkwan University, School of Medicine, Seoul, South Korea; ^9^Department of Laboratory Medicine and Genetics, Samsung Medical Center, Sungkyunkwan University School of Medicine, Seoul, South Korea; ^10^Division of Infectious Diseases, Department of Internal Medicine, School of Medicine, Kyungpook National University, Daegu, South Korea

**Keywords:** COVID-19, SARS-CoV-2, antibody, serology, healthcare worker

## Abstract

The safety of healthcare workers (HCWs) against severe acute respiratory syndrome virus 2 (SARS-CoV-2) transmission is an important aspect of managing the coronavirus disease 2019 (COVID-19) pandemic. In the South Korea, highly stringent infection prevention and control (IPC) guidelines are implemented, and reports of healthcare-associated SARS-CoV-2 transmission among HCWs are limited. However, subclinical infections may have been missed by the current symptom-based screening strategy. To evaluate the risk of undetected SARS-CoV-2 transmissions from COVID-19 patients to HCWs, we conducted a multicenter seroprevalence study after the first surge of the COVID-19 outbreak. A total of 432 HCWs were evaluated, comprising 309 HCWs designated to laboratory-confirmed COVID-19 patient care and 123 non-designated HCWs. Designated HCWs wore personal protective equipment including an N95 respirator, eye protection, hooded overalls, shoe covers, and inner and outer gloves. Use of a powered air-purifying respirator was recommended for aerosol-generating procedures or long-duration care activities. A high-sensitivity (99.1%) fluorescence immunoassay immunoglobulin G (IgG) kit was used as the initial screening test, and two enzyme-linked immunosorbent assay kits for total and IgG antibodies were used to confirm the test results. A microneutralization test was additionally performed to evaluate the neutralizing activity of positive specimens. Among the evaluated HCWs, none of the non-designated HCWs had a positive result, while one of the HCWs designated for COVID-19 patient care (1/309, 0.3%) was seropositive for SARS-CoV-2 with confirmed neutralizing activity (1:40). This finding suggests that subclinical seroconversion may occur among HCWs caring for COVID-19 patients, although the risk is low under strict IPC guidance.

## Introduction

For over 6 months, healthcare workers (HCWs) have been on the frontlines of the coronavirus disease 2019 (COVID-19) pandemic (World Health Organization [WHO], 2020b). Protecting HCWs from severe acute respiratory syndrome virus 2 (SARS-CoV-2) transmission is essential not only to preserve the manpower needed to care for COVID-19 patients but also to prevent in-hospital transmission through HCWs. Because the virus’s mode of transmission and infectivity have not been clearly identified at the beginning of the COVID-19 pandemic, infection prevention and control (IPC) guidelines for HCWs vary across institutions and countries (Kim et al., 2015; CDC, 2020; KCDC, 2020; Liu et al., 2020; World Health Organization [WHO], 2020a). The South Korea introduced highly stringent IPC guidance based on HCW transmission during the 2015 Middle East respiratory syndrome (MERS) outbreak (Kim et al., 2015; Park et al., 2016; Ko et al., 2017; KCDC, 2020) compared with the World Health Organization (WHO) and United States Centers for Disease Control and Prevention (CDC) recommendations for flexible guidelines considering personal protective equipment (PPE) shortages (CDC, 2020; World Health Organization [WHO], 2020a). Screening strategies for COVID-19-designated HCWs can vary according to situation; however, symptom-based real-time polymerase chain reaction (RT-PCR) testing is widely used to detect SARS-CoV-2 infection. However, asymptomatic or subclinical SARS-CoV-2 infections, which are thought to represent a large proportion of infections, could be missed by symptom-based RT-PCR screening strategies (Rivett et al., 2020). Recent evidence showed that asymptomatic and subclinical COVID-19 patients produced detectable amounts of anti-SARS-CoV-2 antibodies, suggesting that subclinical infection could be screened by serologic tests (Ko et al., 2020). To evaluate the risk of undetected SARS-CoV-2 transmission among HCWs caring for COVID-19 patients, we conducted a multicenter seroprevalence study after the first COVID-19 outbreak surge in the Republic of Korea.

## Methods

### Study Population and Personal Protective Equipment Composition

#### Study Population

Healthcare workers from six university hospitals were included: Samsung Medical Center (SMC), Chonnam National University Hospital (CNUH), Chonnam National University Bitgoeul Hospital (CNUBH), Kyungpook National University Hospital (KNUH), Keimyung University Dongsan Hospital (KUDH), and Keimyung University Daegu Dongsan Hospital (KUDDH). KNUH, KUDH, and KUDDH are located in Daegu city. KUDDH was designated for COVID-19 patient care after an outbreak associated with a religious group around the Daegu metropolitan city in February 2020; the entire hospital was used to care for over 1,000 laboratory-confirmed patients for an approximately 2-month period (Korean Society of Infectious Diseases et al., 2020). Other hospitals operated airborne infection isolation rooms (AIIRs) for moderate to severe COVID-19 patients.

Healthcare workers who were designated to COVID-19 patient care were recruited to participate in this study, and those who agreed to undergo serum sampling were enrolled. Non-designated HCWs were also eligible to participate. For designated HCWs, serum sampling was conducted during their time performing COVID-19 patient care or within 1 month of treating their final patient. Patient sampling occurred from late April to early May 2020 and included a simple questionnaire about demographics and symptom experience. Informed consent was obtained from all participating HCWs. The study was approved by the Institutional Review Board (IRB) of SMC (IRB No. SMC 2020-04-066).

#### Personal Protective Equipment Composition

Designated HCWs for COVID-19 patient care wore PPE, including an N95 respirator, eye protection (face shield or goggles), hooded overalls, shoe covers, and inner and outer gloves. Use of a powered air-purifying respirator (PAPR) was recommended for aerosol-generating procedures or long-duration care activities. PPE composition was equivalent to that used for MERS patient care (Kim et al., 2015; Ko et al., 2017).

### Screening Strategy and Test Methods for Anti-SARS-CoV-2 Antibodies

#### Screening Strategy

To screen for the production of anti-SARS-CoV-2-binding antibodies among HCWs, we used a fluorescence immunoassay (FIA) kit (AFIAS COVID-19 Ab assay, Boditech Med Inc., Chuncheon, South Korea). In a previous study, FIA immunoglobulin G (IgG) showed considerable sensitivity (98.8%) for a range of conditions, including convalescent sera of asymptomatic patients to severe laboratory-confirmed COVID-19 patients (Ko et al., 2020). In an upcoming study using 110 RT-PCR-positive and 119 control samples, the sensitivity and specificity were 99.1 and 94.1%, respectively (data unpublished). To exclude non-specific reactions, IFA IgG-positive samples were tested with two enzyme-linked immunosorbent assay (ELISA) kits targeting anti-SARS-CoV-2 total antibody (PCL COVID-19 Total Ab EIA test, PCL Inc., Seoul, South Korea) and IgG antibody (Anti-SARS-CoV-2 ELISA IgG, EUROIMMUN, Lübeck, Germany). Neutralizing activities of ELISA-positive samples were measured by microneutralization (MN) tests.

#### Fluorescence Immunoassay Immunoglobulin G Test

The FIA IgG kit used is based on the automated fluorescent lateral flow immunoassay method using the AFIAS-6 analyzer system (Ryu et al., 2018). This assay uses a sandwich immunoassay with detector SARS-CoV-2 proteins (recombinant nucleocapsid proteins with europium chelate) in a buffer and captures mouse anti-human IgG monoclonal antibodies that are immobilized on a nitrocellulose membrane. After formulating and capturing the antigen–antibody complex on the membrane, time-resolved fluorescence intensity is measured by a scanner and expressed as a relative cut-off index (COI). Specimens with COI value ≥1.1 were considered positive. Whole blood, serum, and plasma specimens can all be used in the assay. All procedures were performed according to the manufacturer’s instructions.

#### ELISA Total Antibody Test

The ELISA total antibody kit detects total antibody against nucleocapsid proteins and the receptor binding domain (RBD) of the SARS-CoV-2 spike protein using the sandwich immunoassay method. In unpublished performance data from 110 RT-PCR-positive and 119 control samples, the sensitivity and specificity of the ELSIA kit were 98.2 and 100%, respectively. All tests were performed in duplicate according to the manufacturer’s instructions. Optical density (OD) ratio <1.0 was interpreted as negative, ≥0.9 to <1.0 as borderline, and ≥1.0 as positive.

#### ELISA Immunoglobulin G Antibody Test

The ELISA kit detects IgG antibodies against SARS-CoV-2 using the S1 domain of the spike protein including the immunologically relevant RBD (EUROIMMUN, 2020). The manufacturer reports 94.4% sensitivity (using 72 specimens collected after day 10 of illness) and 99.6% specificity (using 1,344 control samples, excluding borderline results) (EUROIMMUN, 2020). All tests were performed in duplicate according to the manufacturer’s instructions. OD ratio <0.8 was interpreted as negative, ≥0.8 to <1.1 was borderline, and ≥1.1 was positive.

#### Serum Neutralization Test (Microneutralization Assay)

To evaluate the neutralization activity of the collected specimens, the MN assay against SARS-CoV-2 (Korean isolate; NMC-nCoV02) was performed in duplicate using 96-well tissue culture microplates (Greiner Bio-One, Kremsmünster, Austria) in a biosafety level 3 facility (Ko et al., 2020). We performed twofold serial dilution of inactivated patient serum starting at a dilution of 1:10 using Dulbecco modified Eagle medium (DMEM), incubated solutions with virus samples of 100 tissue culture infective dose 50 (TCID50) for 1 h at 37°C, and then infected Vero cells with the cultures. After 60 min of incubation, the serum and virus mixture was removed, and DMEM was added to the infected cells. The cells were incubated at 37°C in 5% CO_2_ for 4 days. The supernatants were removed, fixed with 10% formalin solution, and stained with crystal violet to determine the titer. Antibody titers were defined as the highest serum dilution that inhibited cytopathic effect (CPE), and a 1:10 dilution was considered the lowest possible significant titer.

### Statistical Analysis

To compare the characteristics and test results of COVID-19-designated and non-designated HCWs, the Student’s *t*-test was used for continuous variables and the Chi-square or Fisher’s exact test for categorical variables. All *P*-values were two-tailed, and those <0.05 were considered statistically significant. IBM SPSS Statistics version 20.0 (IBM, Armonk, NY, United States) was used for the statistical analyses.

## Results

A total of 671 HCWs including 427 HCWs designated for COVID-19 patient care and 244 non-designated HCWs were invited for the study, and 72.4% of designated (309/427) and 50.4% of non-designated (123/244) HCWs were enrolled (Table 1). HCWs were recruited from several hospitals: SMC (*n* = 78), CNUH (*n* = 44), CNUBH (*n* = 31), KNUH (*n* = 116), KUDH (*n* = 51), and KUDDH (*n* = 112). A few HCWs from KUDH and KUDDH rotated between two hospitals. The COVID-19-designated HCWs were younger than the non-designated HCWs (mean ages: 31.1 and 34.9 years, respectively, *P* < 0.001). Significantly higher proportions of females (84.5 and 75.6%, respectively) and nurses (82.2 and 65.9%, respectively) were among the designated HCWs than the non-designated HCWs. The proportion of HCWs with comorbidities was lower among the designated group (5.9 and 13.0%, respectively, *P* = 0.012). Experience with COVID-19-related symptoms, including fever/chills/myalgia, rhinorrhea/nasal stuffiness, cough/sputum/sore throat, anosmia/ageusia, and diarrhea, were observed in similar proportions in the two groups. Cough/sputum/sore throat were the most common symptoms (10.9%), followed by fever/chill/myalgia (9.0%) and rhinorrhea/nasal stuffiness (6.3%). None of the HCWs experienced anosmia or ageusia. Before the enrollment to this study, RT-PCR for SARS-CoV-2 was tested in 40.1% of designated HCWs and 23.6% of non-designated HCWs, all of which were negative. From screening using the FIA IgG test, specimens from seven non-designated HCWs (5.7%, median COI value = 11.49, range: 2.01–20.44) and 13 designated HCWs (4.2%, median COI value = 9.78, range: 1.17–24.49) yielded positive results. Using ELISA for confirmation, only one specimen from a designated HCW yielded a positive result. The specimen had an ELISA total antibody OD ratio of 11.09 (positive result), ELISA IgG antibody OD ratio of 0.87 (borderline result), and positive neutralization activity with an MN titer of 1:40. The seropositive HCW was a 25-year-old male nurse who treated COVID-19 patients in an intensive care unit (ICU) for more than 3 months without experiencing any COVID-19-related symptoms. He had never previously received RT-PCR test for SARS-CoV-2 because he had no known unprotected exposure to SARS-CoV-2 or experienced PPE breakage during COVID-19 patient care. He was tested for SARS-CoV-2 *via* RT-PCR after confirmation of seropositivity, and his RT-PCR result was negative. No other HCW who worked on the team had a positive RT-PCR or serology test result.

**TABLE 1:** Characteristics and serologic test results of COVID-19-designated HCWs compared with non-designated HCWs.

	Non-designated HCWs	COVID-19-designated HCWs	*P*-value
	*n* = 123	*n* = 309	
Age	34.9 ± 10.9	31.1 ± 7.84	<0.001
Male:female sex	30:93 (24.4:75.6)	48:261 (15.5:84.5)	0.031
Occupation			
Doctor	9 (7.3)	34 (11.0)	0.248
Nurse	81 (65.9)	254 (82.2)	<0.001
Laboratory/radiology technician	33 (26.8)	21 (6.8)	<0.001
Comorbidities^∗^	16 (13.0)	18 (5.9)	0.012
Hypertension	5 (4.1)	4 (1.3)	0.079
Diabetes	0 (0.0)	1 (0.3)	0.715
Current smoker	8 (6.5)	7 (2.3)	0.035
Others	6 (4.9)	6 (1.9)	0.092
COVID-19-related symptoms^∗^	19 (15.4)	58 (18.8)	0.415
Fever/chill/myalgia	10 (8.1)	29 (9.4)	0.681
Rhinorrhea/nasal stuffiness	4 (3.3)	23 (7.4)	0.104
Cough/sputum/sore throat	11 (8.9)	36 (11.7)	0.415
Anosmia/ageusia	0 (0.0)	0 (0.0)	NA
Diarrhea	0 (0.0)	5 (1.6)	0.453
RT-PCR test for SARS-CoV-2	29 (23.6)	124 (40.1)	0.001
Positive RT-PCR result	0 (0.0)	0 (0.0)	NA
Serologic tests for SARS-CoV-2	123 (100)	309 (100)	NA
FIA IgG screening, positive	7 (5.7)	13 (4.2)	0.508
ELISA total antibody, positive	0 (0.0)	1 (0.3)	0.715
ELISA IgG antibody, positive	0 (0.0)	1 (0.3)	0.715
Neutralizing activity	No candidates	1 (0.3)^†^	NA

*Data are expressed as number (%) of patients or mean ± standard deviation. ^∗^Some HCWs had multiple comorbidities and/or COVID-19-related symptoms. ^†^MN titer of 1:40. One person was positive for FIA IgG, ELISA total, ELISA IgG, and MN test. HCW, healthcare worker; SARS-CoV-2, severe acute respiratory syndrome virus 2; COVID-19, coronavirus disease 2019; NA, not applicable; RT-PCR, real-time polymerase chain reaction; FIA, fluorescence immunoassay; ELISA, enzyme-linked immunosorbent assay; MN, microneutralization.*

## Discussion

To date, several SARS-CoV-2 seroprevalence studies among HCWs have been conducted, and the results vary widely according to country and outbreak situation. HCW seroprevalence was reported to be as high as 32.6% in New York City, United States (Mansour et al., 2020). Blood specimens were collected from HCWs with a high risk of aerosolized exposures (HCWs in emergency medicine, critical care, and anesthesiology) between March 24 and April 4, 2020. During that 12-day period, confirmed cases in the United States increased fivefold (from 68,440 to 330,891 cases), suggesting an extremely high burden of local transmission and risk of unprotected exposure. Likewise, the seroprevalence of HCWs in outbreak regions with heavy local transmission was high even in COVID-19-free departments. A seroprevalence study of HCWs who did not provide care to COVID-19 patients conducted in an otolaryngology unit in Brescia, Lombardy, Italy, found a seropositive rate of 8.6% (Paderno et al., 2020). Another study conducted in a neurological center in Hameln-Pyrmont, Germany, reported a seropositive rate of 2.9% (Schmidt et al., 2020). Meanwhile, the seroprevalence of HCWs caring for COVID-19 patients has been reported to be much lower, with most studies reporting <2% seropositivity. A seroprevalence study evaluating COVID-19 ICU HCWs at a teaching hospital in New Jersey, United States, reported 0.83% seropositivity (Mughal et al., 2020). In a university hospital in Essen, Germany, the seropositive rate was 1.2% among HCWs treating COVID-19 patients but was higher (5.4%) in HCWs not treating COVID-19 patients (Korth et al., 2020). The lowest seroprevalence among published studies evaluating COVID-19-designated HCWs was 0% (Liu et al., 2020). A total of 420 HCWs from two university hospitals in Guangzhou, Guangdong Province, China, were deployed to Wuhan, China, and designated to COVID-19 patient care. These HCWs received training for PPE use and stayed in designated hotels to minimize personal contact with others. After their 6–8-week deployment, the HCWs were tested *via* RT-PCR and serologic tests, and all tests were negative. These findings suggest that the risk of SARS-CoV-2 transmission in HCWs is higher for community-based transmission or unprotected hospital exposure than among HCWs designated to care for confirmed COVID-19 patients if wearing appropriate PPE.

Importantly, this study found very low seroprevalence (0.3%) of COVID-19 among HCWs caring for COVID-19 patients during an outbreak situation. After an in-hospital outbreak of MERS in 2015, IPC guidelines for emerging respiratory infections became stringent for the South Korea (Kim et al., 2015). At the early phase of the MERS outbreak, recommended PPE consisted of gloves, gown, an N95 respirator, and eye protection (Park et al., 2016). A hooded coverall or PAPR was recommended only for aerosol-producing procedures. However, there were transmission events among MERS-caring HCWs, and contamination of exposed head surfaces was the suspected route of MERS-CoV transmission. After those events, routine use of a hooded coverall was recommended instead of a gown. Designated HCWs were separated from non-MERS patient care. During the COVID-19 pandemic, similarly stringent IPC guidelines have been applied in Asian countries that experienced a SARS outbreak (Liu et al., 2020). However, PPE supply shortages are a concern during a pandemic situation, and such stringent IPC guidance should not be universally recommended (CDC, 2020; World Health Organization [WHO], 2020a). Considering the low seroprevalence among designated HCWs in this study and among deployed HCWs in a study from China, stringent IPC guidance should be considered where applicable. This also emphasizes the importance of preparedness activities including PPE stockpiling and HCW training to meet the stringent IPC guidance not only for the next surge of COVID-19 pandemic but also for the potential future outbreak of another communicable disease.

For screening and confirmation of anti-SARS-CoV-2-binding antibody, we used a two-step protocol that included the FIA IgG kit and two ELISA kits for total and IgG antibody. In a previous study, the FIA IgG kit showed considerable sensitivity (98.8%) even in asymptomatic COVID-19 patients (Ko et al., 2020). The sensitivity of the FIA IgG kit was similar to or higher than that of ELISA kits (98.2% for total antibody and 94.4% for IgG), while ELISA kits were more specific (100% for total antibody and 99.6% for IgG) than the FIA IgG kit (94.1%). Multistep approaches combining methods have been widely used for seroprevalence studies to increase both sensitivity and specificity (Müller et al., 2015; Ko et al., 2017; EUROIMMUN, 2020). The result of the only seropositive specimen of the present study reflects test kit performance. The specimen had a relatively high COI value of 20.85 from the FIA IgG kit (in the previous study, the COI value for positive specimens ranged from 2.3 to 27.8) and an OD ratio of 11.09 from the ELISA total antibody kit (reported range: 1.02–14.83), while the ELISA IgG antibody kit yielded only a borderline OD ratio (0.87) (Ko et al., 2020). A positive neutralizing activity of 1:40 by the MN test, similar titer to those in other asymptomatic or mild symptomatic patients (Ko et al., 2020), indicates that the positive results from the immunoassay kits were true positives. Meanwhile, 19 specimens tested positive by FIA IgG and negative by ELISA. Because ELISA is not a gold standard method, the possibility of false negative of ELISA could not be excluded. However, considering relatively low FIA IgG COI values (8.55 in median) and higher specificity of ELISA, these FIA-positive and ELISA-negative specimens are likely to be negative. Interpretation of seroprevalence studies should be conducted with consideration of kit performance.

This study has several limitations. First, because the purpose of this study was to evaluate overall seroprevalence of COVID-19-designated HCWs, detailed work conditions such as shift times or duties performed were not investigated. Nevertheless, because the applied IPC guidelines were identical across hospitals and only one HCW was seropositive, comparison between seropositive and seronegative HCWs would not be meaningful. Second, this study evaluated seroprevalence at a single time point, and the effect of long-term patient care should be evaluated in a follow-up investigation. Third, we confirmed the presence of binding antibodies using two ELISA methods, and the presence of neutralizing antibody was investigated only in ELISA-positive specimens. We applied this stepwise test protocol based on test kit performance (Ko et al., 2020), but we cannot exclude the possibility that ELISA-negative specimens might have been positive for neutralizing antibody. Fourth, SARS-CoV-2 infection of the seropositive HCW was not confirmed by RT-PCR. Because he remained asymptomatic, he was not screened by RT-PCR before the seroprevalence study, and RT-PCR performed after sampling was negative. After virus exposure, it is not clear whether SARS-CoV-2 seroconversion occurs in the absence of active viral replication or shedding. To our knowledge, only one study has reported an instance of seroconversion among HCWs after unprotected exposure to SARS-CoV with negative results from RT-PCR screening (Chen et al., 2020). This issue requires further investigation. Lastly, whether the seropositive HCW was infected with SARS-CoV-2 during the COVID-19 patient care or from the community is not clear. Epidemiologic tracing was not feasible, since the seropositive HCW did not have any COVID-19-related symptom. None of the coworking HCWs or contacts at the community was infected with SARS-CoV-2. Considering that the COVID-19-designated workers had limited contact with people in the community and the seroprevalence of Korean communities was low at the same time period [one of 3,055 persons (0.03%) was seropositive at a nationwide surveillance conducted from April 21 to June 19, 2020] (MOHW, 2020), the possibility of community transmission might not be high.

## Conclusion

In conclusion, in this seroprevalence study of HCWs designated to COVID-19 patient care in the South Korea, only one HCW (0.3%) was seropositive with neutralizing activity. This finding suggests that subclinical seroconversion may occur among HCWs caring for COVID-19 patients, although the risk is low with strict IPC guidance.

## Data Availability Statement

All datasets presented in this study are included in the article.

## Ethics Statement

The studies involving human participants were reviewed and approved by the Institutional Review Board (IRB) of Samsung Medical Center. The patients/participants provided their written informed consent to participate in this study. Written informed consent was obtained from the individual(s) for the publication of any potentially identifiable images or data included in this article.

## Author Contributions

J-HK, HK, H-HC, SJ, and KP contributed to the conceptualization. J-HK, JL, IJ, CC, S-JK, MH, and H-HC contributed to the investigation. JB, S-JP, E-SK, and YC contributed to the laboratory work and methodology. KP contributed to the supervision. J-HK, HK, H-HC, SJ, Y-JK, E-SK, and KP contributed to the writing, review, and editing. All authors have read and agreed on the submitted manuscript.

## Conflict of Interest

The authors declare that the research was conducted in the absence of any commercial or financial relationships that could be construed as a potential conflict of interest.
